# Editorial: Epidemiology of Avian Influenza Viruses

**DOI:** 10.3389/fvets.2019.00150

**Published:** 2019-05-22

**Authors:** Mathilde C. Paul, Timothée Vergne, Paolo Mulatti, Thanawat Tiensin, Irene Iglesias

**Affiliations:** ^1^IHAP, Université de Toulouse, INRA, ENVT, Toulouse, France; ^2^Laboratorio di Sorveglianza epidemiologica, legislazione veterinaria e benessere animale, Istituto Zooprofilattico Sperimentale delle Venezie, Legnaro, Italy; ^3^Department of Livestock Development, Ministry of Agriculture and Cooperatives, Bangkok, Thailand; ^4^Center for Animal Health Research (CISA), INIA, Madrid, Spain

**Keywords:** avian influenza, epidemiology, surveillance, animal health, control strategies, domestic poultry, wild birds

Avian influenza (AI) is a highly contagious viral disease, characterized by an intense circulation in many wild waterbird reservoir populations, with periodical introduction into the domestic poultry sector. AI viruses have been the source of devastating economic losses in the poultry industry over the last three decades and have become a major veterinary and public health concern due to their zoonotic potential (1, 2). Outbreaks caused by highly pathogenic avian influenza (HPAI) viruses have caused serious animal health crises worldwide, such as the high case fatality rates in poultry, the control measures that are applied (massive pre-emptive culling or vaccination) and the consequences of virus detection on the international poultry produce trade.

The most emblematic illustration of this impact was the emergence of the HPAI H5N1 virus in southern China in the mid-1990s, followed by its continental spread across East and Southeast Asia, and the unprecedented epidemics recorded in 2003–2004. More recently (from 2014 to 2017), several subtypes of HPAI (including H5N1, H5N6, H5N8) have emerged in East Asia and spread intercontinentally, stressing the crucial role of this geographical hotspot as a source of new HPAI subtypes (3, 4). The international dimension and the difficulties in effectively controlling these epidemics, highlight the need for more scientific information in relation to the epidemiology and patterns of the disease in affected countries, especially in East Asia, as well as the need for effective policies against HPAI. This Research Topic aims at contributing to fill this gap. It includes 10 papers which supplement the knowledge of the epidemiology of AI and offer new approaches and insights for surveillance and control strategies in various regions of the world (including France, Germany, the USA, Vietnam, Australia, and Indonesia).

Undoubtedly, the rapid and continuous evolution of AI viruses make their surveillance and control particularly challenging. Dhingra et al. collated all emergence events of H5 and H7 HPAI subtypes, reported since 1959, and used spatial and phylogeographical analyses to shed new light on the emergence processes of highly pathogenic strains. An increase in viral reassortment rates and an antigenic diversity found in China and Vietnam emphasizes the need for further research in these HPAI emergence hotspots. Furthermore, the identification of differences in the spatio-temporal patterns and risk factors for HPAI subtypes in Vietnam, as developed by Mellor et al. highlights the challenge of tailoring surveillance and intervention strategies to the epidemiological contexts and subtypes of interest. Control can be particularly challenging in endemic areas, such as Indonesia, where multiple HPAI virus subtypes and clades may circulate, as described by Durr et al. The authors illustrate the importance of the seed strain used in vaccine developments and of the antigens used to assess sero-protection of vaccinated flocks, under field conditions.

Another great challenge of HPAI control is the intense circulation of AI viruses in waterfowl populations, which act as a natural reservoir with periodic spill-over to domestic poultry. Globig et al. conducted a detailed examination of the distribution of the HPAI H5N8 cases that were reported in wild or captive birds in Germany (2016–2017). They emphasize the lessons learnt during the epidemic in terms of prevention and control, highlighting substantial gaps in farm biosecurity.

AI management is further complicated by the fact that viruses are able to spread through a large number of transmission routes. Identifying these pathways is key in the development of appropriate prevention and control strategies. Walz et al. described the types of potentially infectious or contaminated materials that are disposed of in different US poultry sectors and suggested that poultry farm garbage management and disposal practices may well-contribute to the spread of HPAI viruses between farms. The potential of airborne transmission was questioned by Scoizec et al. who detected the presence of the AIV genome in some air samples collected up to 110 m outside of infected premises during the French HPAI H5N8 epidemic (2016–2017). Based on these results, the authors stress the challenge of implementing the depopulation of infected farms, without contributing to the airborne diffusion of the virus.

Veterinary epidemiology has an eminently applied nature, generating valuable tools of risk analysis that assist decision-making in the animal health sector. The two studies presented by Scott et al. illustrate the relevance of such approaches in the context of early warning systems in disease-free areas. Scenario tree modeling approaches made it possible to assess the pathways of LPAI exposure, as well as to quantify the risk of LPAI and HPAI spread within and between Australian commercial chicken farms.

Timely information is required to optimize the emergency response during outbreaks. In this regard, the questionnaire developed by Umber et al. based on the experience and lessons learnt during an HPAI outbreak in the USA, provides an essential tool in establishing poultry premises status, and tailoring future outbreak management measures. The heterogeneity of actors and organizations involved in poultry production chains is another challenge that needs to be addressed in the design of appropriate measures for AI. Indrawan et al. used a value chain analysis to establish a theoretical framework that makes it possible to examine biosecurity and HPAI control in Western Java, Indonesia, where the disease remains endemic despite extensive efforts. Their results highlight that a proper understanding of the chain governance structure is vital to improve the effectiveness of HPAI control measures, target the incentives, and design fit-for-purpose interventions.

The papers gathered in this Research Topic provide a broad overview of the challenges posed by the surveillance and control of AI viruses (both low and highly pathogenic) in a wide diversity of epidemiological contexts (from disease-free to endemic situations) in different countries. This Research Topic contributes to generating new insights into the epidemiology of avian influenza, which could be used to inform prevention, surveillance and intervention strategies in domestic poultry.

## Author Contributions

The main text has been redacted by MP, TV, PM, and II. All authors revised and approved the editorial.

### Conflict of Interest Statement

The authors declare that the research was conducted in the absence of any commercial or financial relationships that could be construed as a potential conflict of interest.
